# A Compact Fully Electronically Tunable Memristive Circuit Based on CCCDTA with Experimental Results

**DOI:** 10.3390/mi14081484

**Published:** 2023-07-25

**Authors:** Deniz Ozenli

**Affiliations:** 1 Turkish Air Force Academy, Department of Electronics Engineering, National Defence University, 34334 Istanbul, Turkey; dozenli@itu.edu.tr or dozenli@hho.msu.edu.tr; Tel.: +90-212-663-2490-4566 or +90-212-285-36-47; Fax: +90-212-662-8554 or 90-212-285-35-65; 2 Department of Electronics and Communication Engineering, Istanbul Technical University, 34485 Istanbul, Turkey

**Keywords:** current mode, memristor emulator, pinched hysteresis loop, memductance, ANN, synapsis

## Abstract

This work presents a flux-controlled memristor structure employing a Current-Controlled Current Differencing Transconductance Amplifier (CCCDTA) with a grounded capacitor. The proposed emulator’s invariant and variant parts can be safely adjustable, showing promising characteristics of up to 1.5 MHz operating frequency. Furthermore, there is no need for an additional circuit, switching mechanism or changing the circuit topology for the changing of operation modes. To justify the performance of the emulator with incremental and decremental mode operations, a Monte Carlo and temperature analysis are validated using TSMC 0.18 µm technology under a symmetrical supply voltage of ±0.9 V. Furthermore, the workability of the proposed circuit is tested with commercial elements such as ALD1116, AD844 and LM13700. When compared with other studies, the presented emulator circuit demonstrates promising performance in various features.

## 1. Introduction

In modern technologies, there is a strong need for powerful and resilient memory devices. In addition, the significant increase in artificial intelligence applications which employ various complex neural network structures poses new challenges to design more advanced configurations. In this regard, the fabrication process of memristors by the Hewlett-Packard (HP) company in 2018 has attracted circuit designers to reach novel solutions and implementations owing to its unique characteristics.

Memristive elements have a unique feature which combines memory devices and resistive architectures. However, we are still not aware whether these components are available for commercial usage. This is because they necessitate numerous difficult processes and high production costs during fabrication. Consequently, there is a strong need for the fourth element to enable different facilities and architectures in analog and digital circuit design.

There is also a considerable demand for memory and artificial neural networks (ANNs) based on different emulators, providing the advantages of small chip area and design simplicity. In particular, large numbers of architectures have been proposed using the device physics and operating mechanism presented by Chua in [1,2]. From this point of view, Refs. [3,4] present several synaptic circuits while evaluating their utilization in spiking neural networks. Furthermore, Refs. [5,6,7] introduce some neuromorphic applications based on memristive circuits. This is because the use of memristors as synapses not only enables high connectivity but also may offer high density for the implementations where efficient computing is very crucial. In [8], a floating memristor emulator is proposed utilizing a large number of active devices, such as current conveyors and operational amplifiers. In addition, Refs. [9,10] worked on a long-term memory circuit and neuron-inspired encoder system. However, both studies suffer from a large number of active devices and design complexity in achieving memristive behavior for a wide range of operating mechanisms. In [11], a neuromorphic circuit is developed using CMOS technology. Another work presented in [12] introduces a flux-controlled memristor mechanism constructed using an emulator circuit, including seven operational amplifiers, two multipliers and a trigonometric function converter. Ref. [13] proposes a mem-element device using a switching circuitry to control the mode of operation. The circuit combines large numbers of active devices and resistances as passive elements, whereas [14] presents a memristive circuit based on two VDTAs and a switching mechanism. There is an alternative circuit proposed by [15] for utilization in neuromorphic circuits based on a MOSFET-only design. It may encounter additional nonlinearity issues arising from intrinsic parameters of the MOS devices, while [16] presents incremental/decremental modes of operation with different memristor emulators. However, the proposed mechanism requires different circuitries to transition between incremental and decremental operation modes. In addition, Refs. [17,18] propose incremental and decremental mode operations with different architectures. However, they incorporate a couple of resistances as well as active blocks. Ref. [18] necessitates a different circuit to change operation mode.

Some floating memristor emulators are proposed in [19,20,21]. In [19], there is only an incremental mode of operation with large chip area occupation. Ref. [20] makes use of diodes and additional passive components to reach a pinched hysteresis loop. Ref. [21] incorporates an Operational Transconductance Amplifier (OTA) and additional transistors to have incremental/decremental characteristics. Ref. [22] proposes a grounded memristor emulator based on a Differential Voltage Current Conveyor (DVCC) and single OTA with additional passive resistance and switching mechanism. Ref. [23] makes use of Multi-Output OTA (MO-OTA) structure in a feedback structure to obtain a floating memristance. However, it presents only decremental mode characteristics with high complexity. Ref. [24] uses a Current Backward Transconductance Amplifier (CBTA) with additional high-valued capacitances to construct memristive behavior. Refs. [25,26] contain a switching mechanism to change the mode of operation. Ref. [25] includes a Voltage Differencing Current Conveyor (VDCC), whereas [26] employs grounded and floating resistances with Current Conveyor Transconductance Amplifier (CCTA) to obtain pinched hysteresis. Furthermore, Ref. [27] presents an exemplary memristive circuit incorporating two different active blocks with MOSFET-based electronic resistance. It demonstrates a complex operating mechanism while providing pinched hysteresis loops. Ref. [28] has incremental and decremental modes with additional transistors, a single Voltage Differencing Transconductance Amplifier (VDTA) and resistors, whereas [29] utilizes a Voltage Differencing Inverting Buffered Amplifier (VDIBA) and single OTA for all operation modes. Despite providing floating and grounded operations, Ref. [29] requires multiple switching mechanisms for the transition between incremental and decremental modes. While [30] employs OTA and a buffering amplifier to make floating architecture with a switching mechanism, Ref. [31] brings only incremental memristance behavior in different emulators. Ref. [32] presents a compact memristor architecture, which only brings a power consumption of 7.5 µW. In addition, Ref. [33] gives a compound memristor emulator consisting of Second-Generation Current Conveyors (CCIIs) and a single multiplier. However, they combine switching mechanisms, increasing circuit complexity. Ref. [34] gives an alternative architecture as a grounded-type memristor composed of Multiple Output Operational Transconductance Amplifiers (MO-OTAs) with additional passive components. Although it performs well up to 500 kHz, it brings a large number of transistors with ±2.5 V power supplies, which boosts chip area and power consumption. Some grounded and floating memristor emulators are also proposed in [35,36,37,38]. They employ multiple passive elements to reach memristive behavior, where [35] makes use of a single Differential Voltage Current Conveyor Transconductance Amplifier (DVCCTA). In addition, they have flexible architectures operating well up to higher frequencies. On the other hand, these schemes suffer from switching mechanisms, and they also lack electronical adjustability with the help of control voltages.

Memristors are components based on memory devices and resistive architectures. Nevertheless, the commercial availability of these elements is affected by challenging production processes and high fabrication costs. In this respect, there is a strong motivation for the memristor emulators, which can be controlled electronically and incorporate both incremental and decremental operation modes within a unified architecture. In particular, considering the aforementioned studies in the literature, there is preference for simple architectures that include a small number of passive elements and switching mechanisms to change operation mode.

This paper presents a CCCDTA-element-based, grounded memristor circuit. The presented structure is safely tunable with the help of control voltages. The circuit incorporates an active element with a grounded capacitor. It is essential to emphasize that the fixed and variable memristance parts of the proposed memristor can be adjusted separately using control voltages. In addition, it is crucial to highlight that an additional switching mechanism or different circuit topology is not necessary to reach an incremental or a decremental memristive characteristics. Extensive simulations of the presented memristor emulator are investigated on the basis of Cadence Environment with TSMC0.18µm CMOS technology. Theoretical features are also confirmed by experimental results incorporating commercially available elements, such as ALD1116, AD844 and LM13700.

This work comprises the following parts: The next part describes the operation mechanism of the proposed emulator circuit. It also describes the CCCDTA terminals’ relationships and functional behavior. A mathematical basis is given to investigate frequency and time characteristics of the memristor emulator. Further view presents decremental and incremental memristance behaviors, wherein the fixed and variable parts of the emulator can be adjusted separately. The simulation results show the performance of the presented circuit in the Cadence Environment, using postlayout extractions. Performance evaluations are given with extensive comparisons with other studies in the literature. In addition, experimental verifications are presented using commercially available elements to confirm the theoretical part. The last part concludes the paper with final remarks.

## 2. The Proposed Emulator Circuit

CCCDTA characteristics show similarities to conventional CDTA. In addition, the input parasitic resistances of CCCDTA can be easily adjustable with input bias current. CCCDTA has five terminals, two terminals of which are inputs, and the others are outputs. The input terminals have low impedance. The functional matrix of the CCCDTA is given in Equation (1). It is important to note that an active block of CCCDTA consists of the current difference unit and a transconductance block. In the first part, the input current signal is obtained and sent to the second stage as a voltage signal using a Current Differencing Unit (CDU). This enables the input terminal’s current difference to be obtained at the Z terminal. The second part of the CCCDTA incorporates a Dual-Output Operational Transconductance Amplifier (DO-OTA).

**Figure 1:**
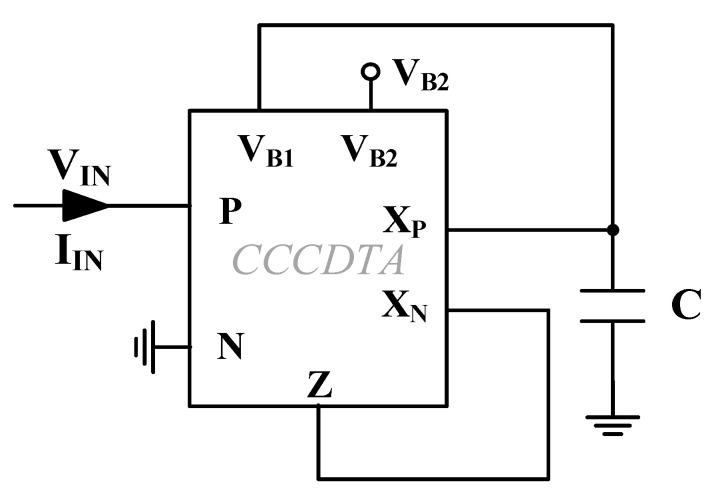
The proposed memristor emulator circuit.

**Figure 2:**
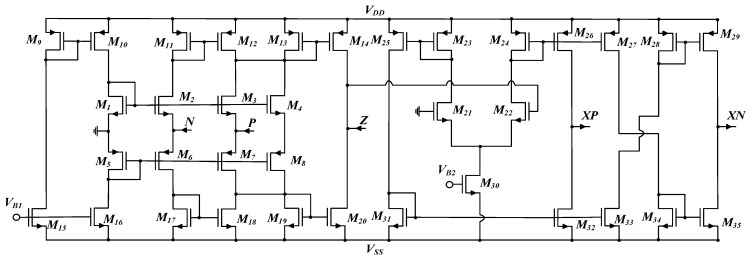
Overall CCCDTA circuit [40].

Considering the functionality of the CCCDTA, the proposed memristor emulator structure can be depicted as given in Figure 1. Based on the circuit implementation of CCCDTA shown in Figure 2, terminal relationships can be derived with the help of the characterization matrix of the CCCDTA.

(1) $$\left[\begin{array}{l} V_{P}\\ V_{N}\\ I_{Z}\\ I_{XP}\\ I_{XN} \end{array}\right]=\left[\begin{array}{ccc} \frac{1}{g_{P}} & 0 & 0\\ 0 & \frac{1}{g_{N}} & 0\\ 1 & -1 & 0\\ 0 & 0 & g_{mp}\\ 0 & 0 & -g_{mn} \end{array}\right]\left[\begin{array}{l} I_{P}\\ I_{N}\\ V_{Z} \end{array}\right]\;\;$$

Equation (1) defines the characterization of the CCCDTA, where *g_P_* and *g_N_* denote the transconductances of the input terminals, respectively. Additionally, *g_mp_* and *g_mn_* denote the output transconductance values. Using this matrix and the current and voltage relationships of the proposed emulator circuit’s terminals illustrated in Figure 1, the following equations can be described as follows:

(2) $$I_{IN}=I_{P}=I_{Z}\;\;\;$$

(3) $$V_{IN}=V_{P}\;\;\;$$

Using the relationship given in (1):

(4) $$I_{Z}=-g_{mn}V_{Z}\;\;$$

(5) $$g_{P}V_{IN}=-g_{mn}V_{Z}\;$$

The *V_Z_* voltage can be obtained as

(6) $$V_{Z}=-\frac{g_{P}}{g_{mn}}V_{IN}\;$$

In this respect, the dropped voltage across the grounded capacitance *V_C_*_,_ can be evaluated in the following relationship, where *V_Z_* voltage is described in (6):

(7) $$V_{B1}=V_{C}=\frac{1}{C}\int I_{XP}dt\;$$

(8) $$V_{C}=-\frac{g_{mp}g_{P}}{g_{mn}C}\int V_{IN}dt\;$$

(9) $$V_{C}=-\frac{g_{mp}g_{P}}{g_{mn}C}\Phi_{IN}\;$$

Using the translinear principle in [39], the Equations (10)–(12) can be solved as follows:

(10) $$g_{P}=2g_{mx}=2K\left(V_{B1}-V_{SS}-V_{TH}\right)\;\;$$

(11) $$g_{P}=2g_{mx}=2K\left(-\frac{g_{mp}g_{P}}{g_{mn}C}\Phi_{IN}-V_{SS}-V_{TH}\right)\;\;$$

Considering the overall CCCDTA circuit given in Figure 1, the conductance value seen from the *P* terminal can be calculated as defined in (11), where *K* is defined as follows:

(12) $$K=C_{ox}\sqrt{\mu_{n}{\left(\frac{W}{L}\right)}_{15}}\left(\sqrt{\mu_{p}{\left(\frac{W_{p}}{L_{p}}\right)}_{7}}+\sqrt{\mu_{n}{\left(\frac{W_{n}}{L_{n}}\right)}_{3}}\right)\;\;\;\;$$

where *g_mx_* = *g_m_*_2,3_ ≅ *g_m_*_6,7_. In this regard, the memductance value can be evaluated as follows:

(13) $$g_{P}=\frac{2K\left(-V_{SS}-V_{TH}\right)}{1+\frac{2g_{mp}K}{g_{mn}C}\Phi_{IN}}\;\;\;$$

(14) $$M\left(\Phi_{IN}\right)=\frac{V_{IN}}{I_{IN}}=\frac{1}{g_{P}}=\underset{\mathrm{fixed}\;\mathrm{part}}{\underbrace{\frac{1}{2K\left(-V_{SS}-V_{TH}\right)}}}+\underset{\mathrm{variable}\;\mathrm{part}}{\underbrace{\frac{g_{mp}\Phi_{IN}}{g_{mn}C\left(-V_{SS}-V_{TH}\right)}}}\;\;$$

(15) $$M\left(\Phi_{IN}\right)=\frac{V_{IN}}{I_{IN}}=\frac{1}{g_{N}}=\frac{1}{2K\left(-V_{SS}-V_{TH}\right)}-\frac{g_{mp}\Phi_{IN}}{Cg_{mn}\left(-V_{SS}-V_{TH}\right)}\;\;\;$$

Here, (14) and (15) provide both incremental and decremental mode operation of the proposed memristance emulator. It is important to note that supply voltage (*V_SS_*) becomes negative-valued, and the time-invariant part (fixed part) becomes positive-valued. Equations (14) and (15) indicate that the fixed part can be varied electronically by the *V_SS_* voltage source, while the time-variant part (variable part) can be safely controlled electronically with the help of *g_mp_*/*g_mn_*.

(16) $$\Phi_{IN}=\frac{V_{AMP}\cos\left(\omega t-\pi \right)}{\omega }\;\;\;$$

If (16) is substituted with (14) and (15), the following equations can be found as

(17) $$M\left(\Phi_{IN}\right)=\frac{V_{IN}}{I_{IN}}=\frac{1}{g_{P}}=\frac{1}{2K\left(-V_{SS}-V_{TH}\right)}+\frac{g_{mp}V_{AMP}\cos\left(\omega t-\pi \right)}{g_{mn}\omega C\left(-V_{SS}-V_{TH}\right)}\;\;$$

(18) $$M\left(\Phi_{IN}\right)=\frac{V_{IN}}{I_{IN}}=\frac{1}{g_{N}}=\frac{1}{2K\left(-V_{SS}-V_{TH}\right)}-\frac{g_{mp}V_{AMP}\cos\left(\omega t-\pi \right)}{g_{mn}\omega C\left(-V_{SS}-V_{TH}\right)}\;$$

In this regard, Equations (17) and (18) describe the incremental and decremental memristance behaviors, respectively.

Considering the memristance emulator circuit in Figure 1, the incremental memristance can be obtained with the help of input voltage applied to the positive terminal of the CCCDTA, whereas decremental behavior can be observed during input signal injection to the negative terminal. The proposed memristor emulator, including CCCDTA, is dimensioned as tabulated in Table 1, where the overall CCCDTA structure is given in Figure 2.

Considering the operating mechanism of the proposed memristor emulator seen in (17) and (18), the time-variant parts of the memristance values can be adjusted by means of *V_B_*_2_. When the ratio of *g_mp_*/*g_mn_* increases, the time-variant parts become dominant, besides the time-invariant parts of incremental and decremental operation modes. Table 2 demonstrates that the ratio of output terminals’ transconductances can be easily modified with the help of *V_B_*_2_ bias voltage of M30, as illustrated in Figure 2.

**Figure 3:**
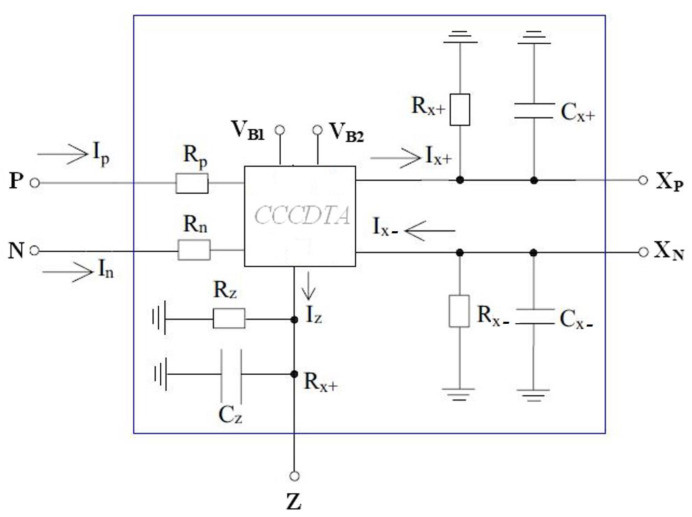
CCCDTA representation with parasitic effects.

The parasitic effects arising from the nonideal behavior of the proposed emulator circuit in Figure 1 can be depicted as illustrated in Figure 3. In the ideal case, R_z_, R_x+_ and R_x−_ are the parasitic resistances that occur in parallel at the terminals z, x+ and x−, respectively. They can be evaluated as infinity. In addition, the parasitic capacitances C_z_, C_x+_ and C_x−_ become zero. On the basis of the parasitic impedances, the capacitor of C employed in the design should usually be quite higher than the parasitic capacitances (C >> C_x+_ and C >> C_x−_), so that parasitic capacitance effects can be minimized around operating frequencies. To reduce the effects of the CCCDTA parasitic resistances worsening the proper memristive behavior, C should be selected under the conditions: 1/sC << R_x+_ and 1/sC << R_x−_. Grounded capacitance of the memristor emulator illustrated in Figure 1 facilitates the usage of MOS-Cap instead of real capacitance. In this regard, chip area occupation can be significantly reduced, allowing for the attainment of the high-frequency nonlinear behavior of the pinch hysteresis loops. However, MOS-Cap employment worsens the aforementioned parasitic effects. The MOS-Cap intrinsic capacitances bring additional nonlinearity with respect to control voltages. Also, the parasitic capacitances seen from CCCDTA’s input terminals can be disregarded for a wide range of frequencies (considering the overall circuit given in Figure 1 and dimensioned in Table 1, the input impedances have resistive characteristics of up to around 450 MHz).

## 3. Simulation Results

**Figure 4:**
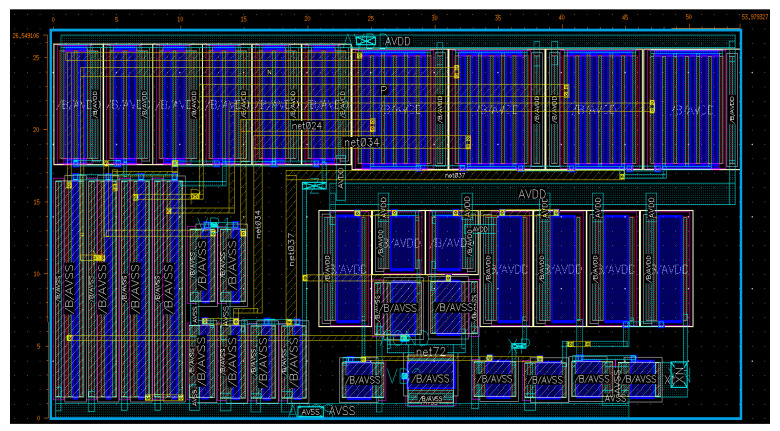
Layout of the overall memristor emulator (26.5 µm × 53.9 µm).

The presented structure has been fully implemented in the Cadence Virtuoso environment using TSMC 0.18 µm CMOS technology under ±0.9 V symmetrical power supplies. All simulations are given on the basis of extracted postlayout simulations as well. The memristor emulator illustrated in Figure 1 is examined through extensive analyses, where NMOS bulk terminals are connected to the most negative voltage and the PMOS devices’ bulks are shorted to the most positive voltage. Figure 4 gives the overall circuit’s layout to evaluate postlayout performance (the total area is around 26.5 µm × 53.9 µm).

Considering the proposed emulator circuit as depicted in Figure 1 in conjunction with Figure 2, V_*B*1_ is selected as −200 mV, and V_*B*2_ is adjusted as −160 mV for the proper bias conditions around the 20 µA drain current of M30. In this regard, terminal resistances of the CCCDTA terminals are given as r_P_ = 686 Ω, r_N_ = 688 Ω, r_Z_ = 156.5 kΩ r_XP_ = 910.4 kΩ and r_XN_ = 906.6 kΩ, respectively. Consequently, the current following ratios of the CCCDTA are found as I_Z_/I_P_ = 0.998 and I_Z_/I_N_ = 0.958 for a wide range of frequencies (up to 450 MHz). Furthermore, the grounded capacitance of the emulator circuit is selected as 10 pF.

**Figure 5:**
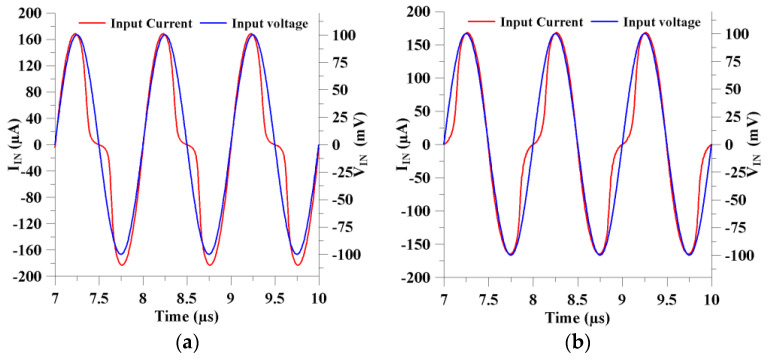
The presented memristor emulator’s time-domain responses: (**a**) incremental and (**b**) decremental mode.

**Figure 6:**
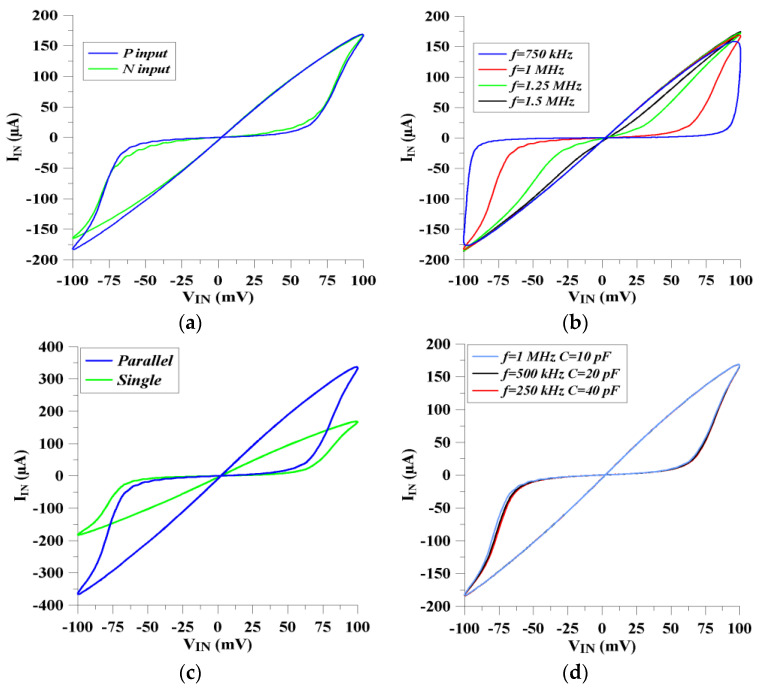
**Memristor characteristics:** (**a**) incremental and decremental modes of the proposed memristor structure (blue is incremental mode and green is decremental mode), (**b**) variation of the hysteresis from 750 kHz up to 1.5 MHz, (**c**) single and parallel connected memristors’ voltage current relationships and (**d**) voltage–current relationship of the proposed memristor emulator for the constant ωC values in decremental mode.

The memristor’s current and voltage signals are investigated with respect to the time axis in Figure 5a,b for incremental and decremental features, respectively. When the injected input voltage equals zero, the memristor current becomes zero as well. The change in the operation modes of the proposed memristor emulator can be seen from Figure 6a, where the input signal injection is applied to the P and N input terminals, respectively. In addition, the memristor has frequency-dependent characteristics so that it behaves as a combination of nonlinear and linear resistive characterization. When applied to high frequencies, it shows linear resistance behavior, while it shows nonlinear resistance behavior at low frequencies. As shown in Figure 6b, the memristor shows nonlinear voltage–current behavior at lower frequencies. However, it becomes more linear-resistant when the input signal frequency increases.

Furthermore, the parallel memristors should have more conductive features in comparison with single memristors. This is because memristors have resistive behaviors. Also, the voltage–current relationship of the parallel memristive devices becomes more nonlinear compared with the single memristive block. Therefore, hysteresis loops are justified and obtained, as shown in Figure 6c. Figure 6d shows an examination of the effect of the capacitor that is employed in the proposed memristor emulator. According to Equations (17) and (18), the voltage–current behaviors should be similar when the multiplication of operating frequency and capacitor value are the same. In Figure 6d, 0.25 MHz, 0.5 MHz, and 1 MHz frequencies are injected into the emulator circuit. Similar hysteresis loops are obtained for the capacitor values of 40 pF, 20 pF and 10 pF, respectively, with 100 mV of input signal amplitude.

**Figure 7:**
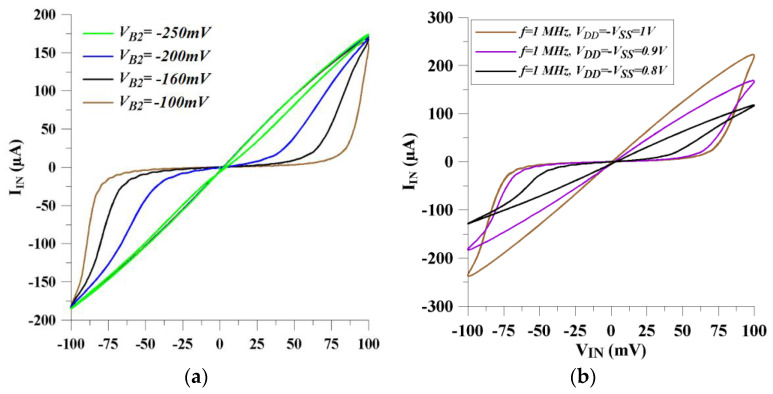
The voltage–current curves for different (**a**) V_*B*2_ bias voltage values and (**b**) *V_SS_* voltage values.

In Figure 7a, the memristor shows more linear behaviors when the control voltage of V_*B*2_ shifts to more negative voltages. As a result, the memristor circuit’s working frequency can be adjusted electronically by altering the V_*B*2_ voltage. Also, the *V_SS_* voltage value influences the memristor performance. Equations (14) and (15) clearly state that *V_SS_* directly affects the average memductance value, namely the time-invariant part of the memductance can be controlled by changing the *V_SS_* voltage. As shown in Figure 7b, the absolute value of the current increases as the *V_SS_* decreases.

**Figure 8:**
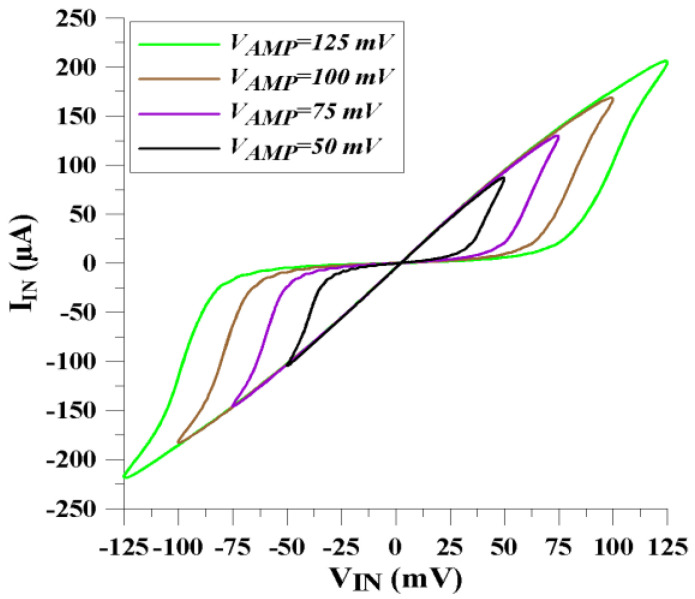
Input signal’s amplitude effect for the memristance behavior.

The time-variant part of the proposed memristor emulator structure is dependent on the amplitude value of the input voltage signal, as shown in Figure 8. This behavior confirms the theoretical structure of the emulator as given Equations (17) and (18).

**Figure 9:**
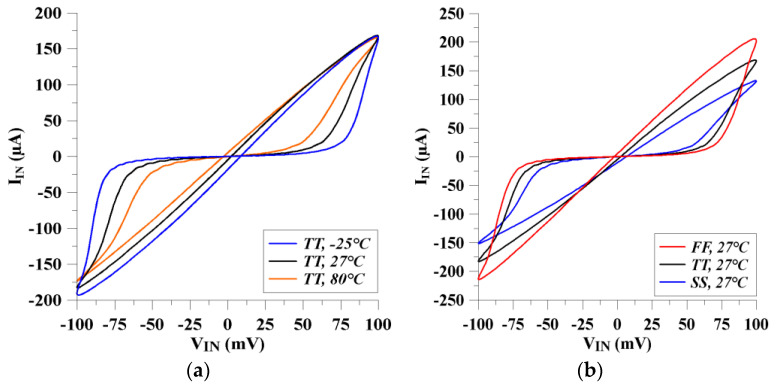
The memristor behavior with respect to different operation conditions: (**a**) the different temperatures and (**b**) process corners.

**Figure 10:**
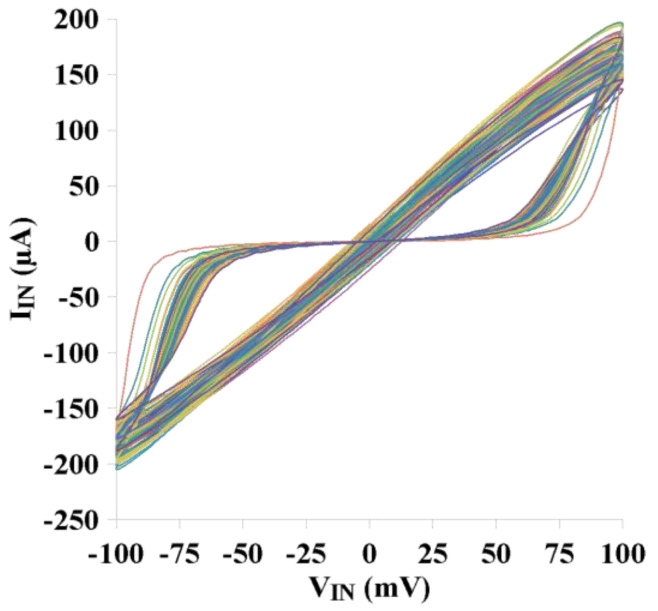
Monte Carlo simulations for the 100 random Monte Carlo seeds.

To verify the performance of the memristor emulator, pinched hysteresis loops are given for various process corners and a wide range of temperatures. As depicted in Figure 9a, slightly changed voltage–current hysteresis loops can be obtained for the wide range of temperatures (−25 °C to 80 °C). From Figure 9a, it is evident that the memristor current increases as the temperature level goes down. Regarding the different process corners, Figure 9b clearly indicates that the memristor emulator circuit still remains within the acceptable region to work in the specified operation mode. Meanwhile, to deepen the analysis of mismatches between the transistors and process corners on the proposed memristor emulator, the Monte Carlo analysis is presented in Figure 10. The Monte Carlo realization is given for a hundred times. The pinched hysteresis loop for the memristor circuit is shifted slightly. The performance of the proposed memristor remains within the acceptable limits.

**Figure 11:**
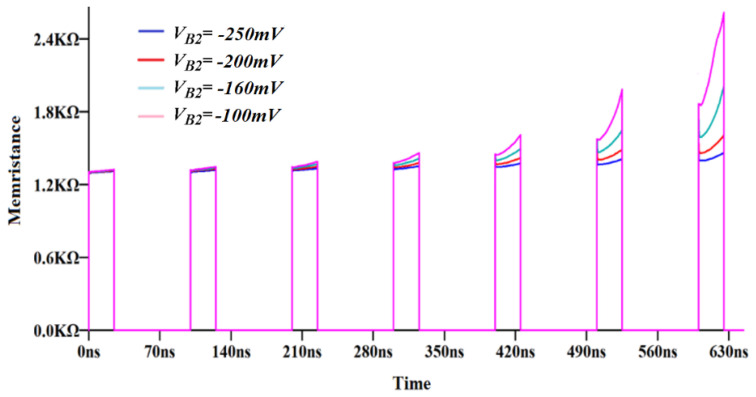
Variation of the incremental memristance value (V_*B*2_ varies between −250 mV and −100 mV).

As given in Figure 11, the memristor current decreases within each pulse. In this memory verification, pulse duration is selected as 25 ns, whereas the period is 100 ns. The current decreases in the subsequent pulses, whereas the memristance remains unchanged in the interval of the pulses. It is important to note that the proposed memristor emulator circuit operates as a memory device. Figure 11 points out the effect of V_*B*2_ voltage on the average memristance value. By adjusting V_*B*2_ voltage to the more negative voltages, the average memristance value decreases. The proposed memristor shows promising performance in comparison with previous studies, as depicted in Table 3. The table presents information on the number of active and passive elements, electronically controllable property and conditions for changing the operation modes.

The proposed emulator circuit can be justified based on a first-order high-pass filter structure. To adjust the roll-off frequency of the filter, it should be considered that the memristor behavior can be controlled electronically. Also, the operation mode of the memristance affects the filter’s gain and cut-off frequency according to the following equations between (19) and (21) [41]:

(19) $$R_{M}=R_{average}\pm \Delta R_{M}\cos(\omega t-\phi )$$

(20) $$Gain(dB)=-10\log_{10}\left(\left(R_{average}\pm \Delta R_{M}\cos(\omega t-\phi )\right)\cdot C_{filter}\right)$$

(21) $$f_{-3dB}=\frac{1}{2\pi R_{M}C_{filter}}$$

where *R_M_* denotes the memristance value that is affected by the amplitude of sinusoidal resistance changing across *R_average_*, while ϕ represents the phase shift in the input signal. *C_filter_* gives the capacitance value used in the first-order high-pass filters. In this respect, the average and changing parts can be evaluated based on Equations (17) and (18) as follows:

(22) $$R_{average}=\frac{1}{2K\left(-V_{SS}-V_{TH}\right)}$$

(23) $$\Delta R_{M}=\frac{g_{mp}V_{AMP}}{g_{mn}\omega C\left(-V_{SS}-V_{TH}\right)}$$

**Figure 12:**
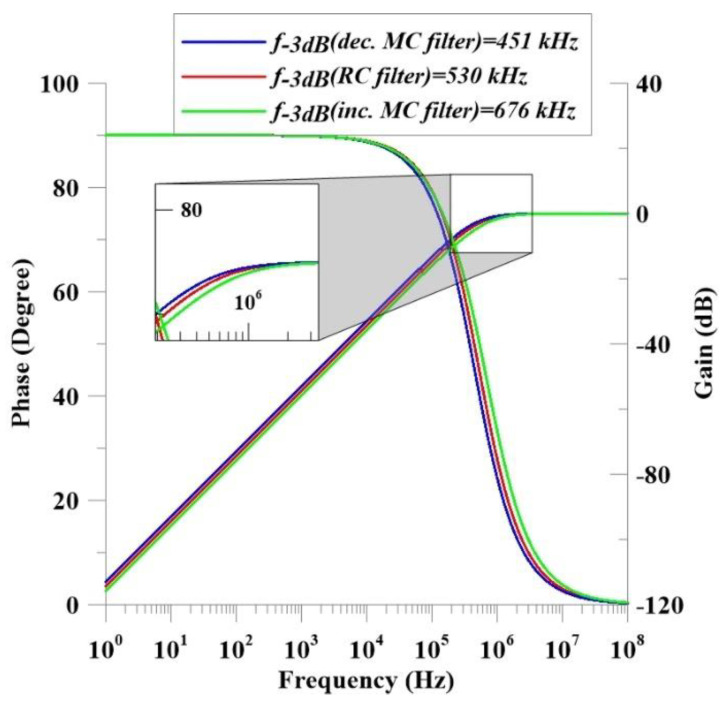
The frequency response of the proposed memristor-emulator-based high-pass filters: decremental MC filter, incremental MC filter and RC filter.

As shown in Figure 12 and Equations (19)–(23), the incremental and decremental configurations have different cut-off frequencies in the first-order high-pass filter application, where *C_filter_* is selected as 50 pF. In the incremental mode, the cut-off frequency is 676 kHz, while the decremental mode brings the cut-off frequency to around 450 kHz. In addition, the memristance value can be evaluated as 4.6 kΩ in the incremental configuration, while this value is around 7 kΩ in the operation of the decremental mode. This variation results from changing part of the memristance value in Equation (23) with regard to different operation modes.

**Table 3:** Previous architectures from the literature in comparison with the present work.

Reference	Active Elements	Passive Elements	Number of Transistors	Actual Layout Area/Approximated Layout Area **	Floating or Grounded	Power Consumption	Electronically Adjustability	Required Conditions to Reach Incremental and Decremental Modes
[12] *	1 OPAMP, 1 Multiplier, 1 Sine Converter	16 Resistors, 1 Capacitor	NA	NA	Grounded	>5 mW	Fixed and variable parts	Only incremental mode
[13]	3 AD844 and 1 Multiplier	6 Resistors	NA	NA	Floating	850 mW	×	Usage of the multiple switching mechanisms
[15]	10 Transistors	2 Capacitors	10	NA/55 µm^2^	Floating	NA	NA	NA
[16]	1 OPAMP	3 Resistors, 1 Capacitor	NA	NA	Floating	NA	×	Usage of the multiple switching mechanisms
[17]	1 DDCC, 1 Multiplier	2 Resistors, 1 Capacitor	50	NA/645 µm^2^	Floating	NA	×	Usage of different circuit topology
[18]	4 AD844 and 1 AD633	3 Resistors, 1 Capacitor	NA	NA	Floating	NA	×	Usage of different circuit topology
[19]	4 AD844 and 1 AD633	4 Resistors, 1 Capacitor	NA	NA	Floating	>1 mW	×	Circuit topology remains the same
[20]	4 AD844 and 2 1N4148	4 Resistors, 4 Capacitors	NA	NA	Floating	>1 mW	×	NA
[21]	1 OTA and 3 Transistors	2 Capacitors	NA	NA	Floating	>1 mW	NA	Circuit topology remains the same
[22]	1 OTA and 1 DVCC	1 Resistor, 1 Capacitor	23	NA/130 µm^2^	Grounded	NA	NA	Usage of the switching mechanism
[23] *	1 MO OTA	1 Capacitor	17	476 µm^2^/136 µm^2^	Floating	NA	Fixed and variable parts	Only decremental mode
[24]	1 CBTA	2 Capacitors	31	NA/185 µm^2^	Floating	NA	×	Circuit topology remains the same
[25]	1 VDCC and 2 Transistors	1 Capacitor	26	1160 µm^2^/100 µm^2^	Grounded	NA	Fixed and variable parts	Usage of the switching mechanism
[26]	1 CCTA	3 Resistors, 1 Capacitor	30	NA/311 µm^2^	Grounded	>7.5 mW	×	Usage of the switching mechanism
[27]	1 DO-OTA,1 DVCC, 2 Transistors	1 Capacitor	29	10,700 µm^2^/416 µm^2^	Floating	>1 mW	Fixed and variable parts	Circuit topology remains the same
[28]	1 VDTA, 19 Transistors	2 Resistors, 1 Capacitor	19	NA/121 µm^2^	Floating	1.34 mW	×	Usage of the switching mechanism
[29]	1 VDIBA, 1 OTA, 2 Transistors	1 Resistor, 1 Capacitor	20	624 µm^2^/57 µm^2^	Floating	NA	Fixed and variable parts	Usage of the multiple switching mechanism
[30]	1 OTA, 1 VDBA	1 MOS-Cap	25	12,075 µm^2^/NA	Floating	NA	×	Usage of the switching mechanism
[31] ^a^*	1 OTA, 1 VF, 1 Transistor	×	16	NA/77 µm^2^	Grounded	NA	×	Only incremental mode
[31] ^b^*	1 OTA, 1 Transistor	×	10	NA/162 µm^2^	Grounded	NA	×	Only incremental mode
[32]	1 DVCC, 3 Transistors	×	15	442 µm^2^/50 µm^2^	Grounded	7.5 µW	Only Variable Part	Usage of the switching mechanism
[33]	2 CCII, 1 Multiplier	2 Resistor, 1 Capacitor	NA	NA	Floating	NA	x	Usage of the multiple switching mechanism
[34]	4 MO-OTA	3 Resistor, 1 Capacitor	92	NA/495 µm^2^	Grounded	NA	Fixed and variable parts	Usage of the switching mechanism
[35]	1 DVCCTA	3 Resistor, 1 Capacitor	28	NA/244 µm^2^	Grounded	NA	×	Usage of the switching mechanism
[36]	1 CCII, 1 OTA	1 Resistor, 1 Capacitor	24	5250 µm^2^/145 µm^2^	Grounded	9.5 mW	×	Usage of the switching mechanism
[37]	1 CCII, 1 MO-OTA	1 Resistor, 1 Capacitor	30	4829 µm^2^/179 µm^2^	Floating	9.8 mW	×	Usage of the switching mechanism
[38]	1 CCII, 1 CCTA	3 Resistor, 1 Capacitor	38	NA/421 µm^2^	Floating	NA	×	Usage of the switching mechanism
**The Present Work**	1 CCCDTA	1 Capacitor	35	1428 µm^2^/265 µm^2^	Grounded	715 µW	Fixed and variable parts	Circuit topology remains the same

* The cited work presents only the incremental mode of memristive behavior. NA represents that there is no information regarding the related parameter. VF symbolizes voltage follower. ** Approximated layout area is calculated by summing the products of the channel widths and channel lengths of each transistor, where there is no information about the exact layout area. ^a^ The first architecture in the related work. ^b^ The second architecture in the related work.

The proposed memristor provides an alternative solution compared with previous studies, as shown in Table 3. The table provides information on the number of active and passive elements, electronically controllable property, power consumption, number of transistors and conditions for the change in operation modes. Considering the different architectures in the previous works, multiplier circuits or a large number of active elements and floating components have been employed to acquire more nonlinear behavior, resulting in the requirement of more power consumption and large chip area. From this perspective, this work proposes a compact architecture without any multiplier blocks to obtain the nonlinear characteristics of the memristor. It is noteworthy that some of the proposed architectures have a floating architecture. However, they bring switching mechanisms or need different topologies to change the operation mode. Unlike in most previous studies, the proposed memristor emulator allows for safe adjustment, enabling the separate control of the invariant and variant parts through control voltages.

## 4. Experimental Verifications

**Figure 13:**
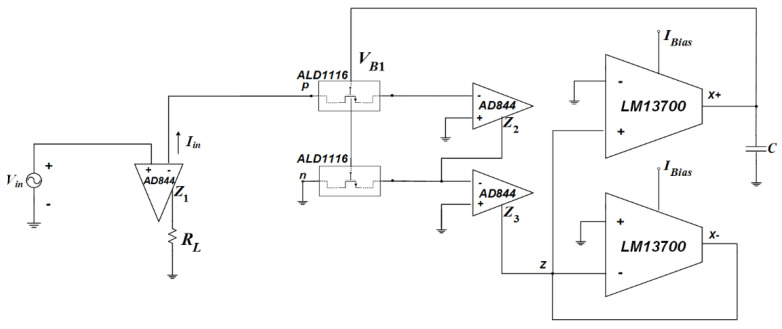
Practical measurement set-up of the proposed memristor emulator based on commercial off-the-shelf elements.

The performance of the memristor emulator structure is confirmed with the help of commercial off-the-shelf elements employed based on CCCDTA structure, as shown in Figure 13. In the measurement test bench, all integrated circuits are operated under power supplies of ±10 V. Additionally, the bias currents of the LM13700 are generated using bias resistances with a value of 56 kΩ, whereas the capacitance and load resistance are valued at 100 nF and 2.2 kΩ, respectively. To obtain input current, the current output terminal of the AD844 is used. In addition, to alter the input terminal resistances of the CCCDTA, the capacitor voltage is utilized. Tunable resistances operated in the triode region can be easily obtained using MOS devices incorporated by ALD1116. Furthermore, an additional level shifter circuit can be employed to keep the MOS devices in the deep triode region.

**Figure 14:**
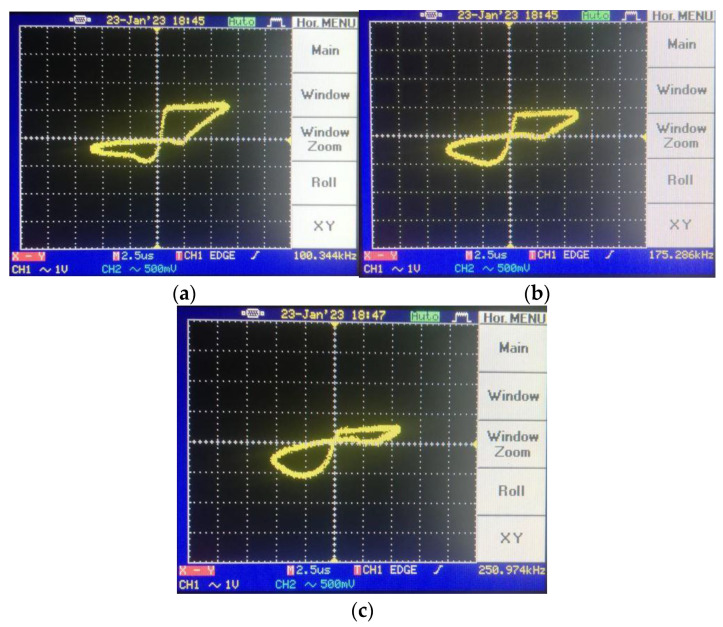
Pinched-loop hysteresis measurement for different frequencies in incremental mode: (**a**) @100 kHz hysteresis, (**b**) @175 kHz hysteresis and (**c**) @250 kHz hysteresis.

**Figure 15:**
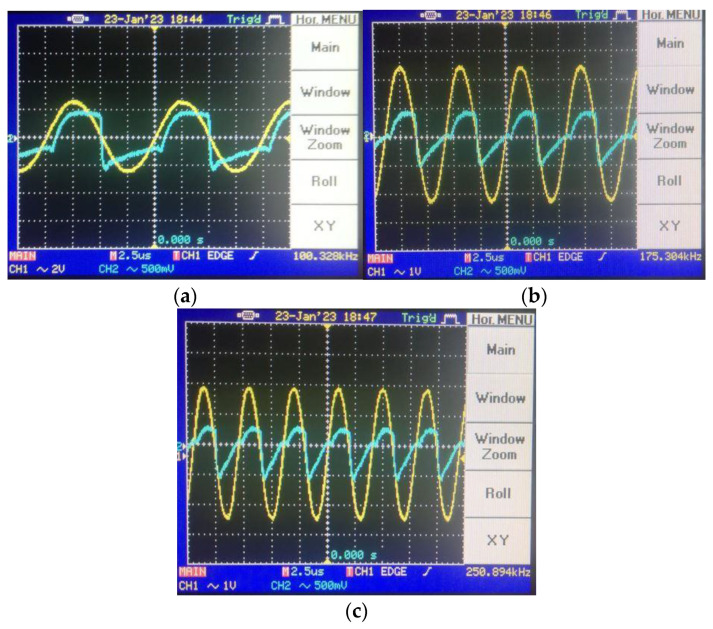
Transient responses for different frequencies in incremental mode: (**a**) @100 kHz transient, (**b**) @175 kHz transient and (**c**) @250 kHz transient (in the transient response, the yellow color output is input voltage, whereas the blue color one illustrates the load resistance’s voltage).

**Figure 16:**
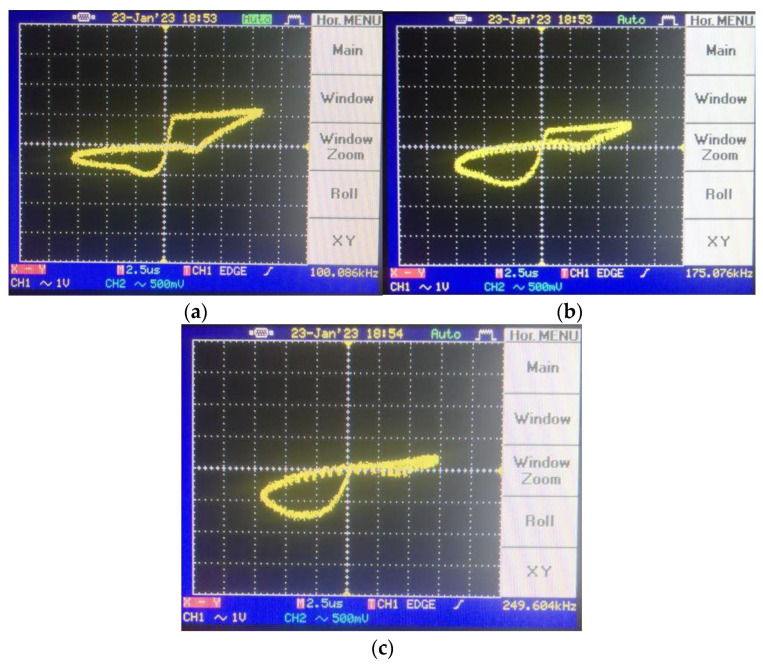
Pinched-loop hysteresis measurement for different frequencies in decremental mode: (**a**) @100 kHz hysteresis, (**b**) @175 kHz hysteresis and (**c**) @250 kHz hysteresis.

**Figure 17:**
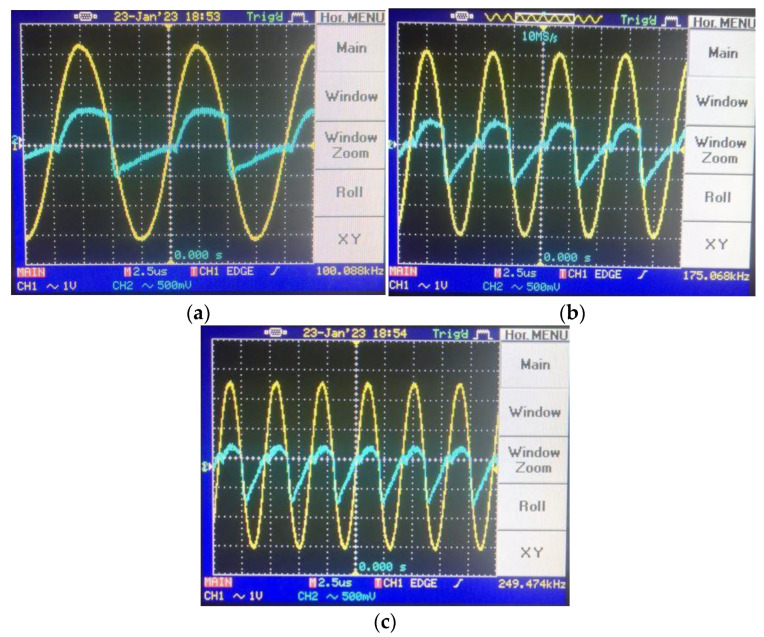
Transient responses for different frequencies in decremental mode: (**a**) @100 kHz transient, (**b**) @175 kHz transient and (**c**) @250 kHz transient (in the transient response, the yellow color output is input voltage, whereas the blue color one illustrates the load resistance’s voltage).

To shift the operation mode of the memristor emulator from incremental to decremental, the input voltage should be shorted to the n terminal. Figure 14 gives the pinched hysteresis loops depicting the current–voltage relationship with respect to input signal frequency, where the peak-to-peak input amplitude is around 4 V. Figure 15 shows transient responses in each case of the incremental mode operation. In accordance with the theoretical aspect, when input signal frequency increases, the memristor emulator exhibits more linear behavior, owing to the decreasing time-variant part. Figure 16 illustrates decremental memristive behavior with respect to increasing operating frequency, while Figure 17 presents transient responses in the decremental mode. However, it is crucial to consider that the limited bandwidth of the integrated circuits and additive noisy operating conditions might be worsening measurement conditions.

## 5. Conclusions

This work presents a CCCDTA-element-based, grounded memristor circuit. The proposed structure is safely tunable with the use of control voltages, where the circuit incorporates a single active element with a grounded capacitor. It is critical to point out that the fixed and variable memristance parts of the proposed memristor can be adjusted separately using control voltages. It is also important to note that an additional switching mechanism or different circuit topology is not required to reach incremental or decremental memristive characteristics. Extensive simulations of the presented memristor emulator were performed using TSMC 0.18 µm CMOS technology.

Taking into account the different architectures in previous works, multiplier circuits or a large number of active elements and floating components have been utilized to acquire more nonlinear behavior so that more power consumption and a large chip area are necessitated. From this view, this work presents a compact architecture without any multiplier blocks to reach the nonlinear characteristics of the memristor. It should be noted that some of the proposed architectures have a floating architecture. However, they bring switching mechanisms or need different topologies to change the operation mode. Unlike in most previous studies, the proposed memristor emulator allows for safe adjustment, enabling the separate control of the invariant and variant parts through control voltages.

The memristor emulator circuit that incorporates a single active and a grounded passive element is presented based on postlayout simulations. The different emulators proposed in previous works make use of multiplier circuits in order to acquire more nonlinear behavior, or a lot of active elements and floating components are used, so that they require more chip area and huge amounts of power. In summary, the proposed circuit outperforms the previous memristor emulator architectures in terms of some important features. First, the grounded memristor structure is obtained from the circuit without modifying the circuit structure or using an additional switching mechanism to obtain different operation modes. Second, unlike in many previous studies, the memristance value of the proposed circuit can be adjusted electronically. It can also be tunable through the use of bias voltages in both the time-invariant and variant parts. As the final point, the circuit can be implemented using a single active element and a grounded capacitance. The postlayout performance is given with Monte Carlo realizations, temperature variations and process corners. All simulation results are presented in extensive comparisons with previous studies. Finally, experimental verifications justify the operation of the proposed memristor emulator using commercially available ICs.

## Figures and Tables

**Table 1:** Dimensions of the transistor in CCCDTA.

Transistors	W (μm)	L (μm)
M_1_–M_4_	15	0.36
M_5_–M_8_	45	0.36
M_9_–M_14_	15	0.5
M_15_–M_20_	5	0.5
M_21_–M_24_, M_30_	3.6	1.8
M_25_–M_29_	7.2	1.8
M_31_–M_35_	2.4	1.8

**Table 2:** CCCDTA output terminals’ transconductance variation versus bias voltage of V_*B*2_.

**V_*B*2_ (mV)**	−250	−200	**−160**	−130	−100
***g_mp_* (μA/V)**	114.8	146.8	**170.5**	187.3	199.8
***g_mn_* (μA/V)**	115	146.1	**169.3**	185.6	197.6
**Emulator Power Consumption (µW)**	615	661	**715**	748	780

## Data Availability

It is clarified that data sharing is not applicable to this article because there are no specifically produced datasets in the presented work.
